# The Nature of Phenotypic Variation in Pavlovian Conditioning

**DOI:** 10.1037/xan0000177

**Published:** 2018-10

**Authors:** Adela F. Iliescu, Jeremy Hall, Lawrence S. Wilkinson, Dominic M. Dwyer, R. C. Honey

**Affiliations:** 1 School of Psychology, Cardiff University; 2 School of Medicine, Cardiff University; 3 Schools of Psychology and Medicine, Cardiff University; 4 School of Psychology, Cardiff University

**Keywords:** rat, sign-tracking, goal-tracking, contingency, associative structures

## Abstract

Pavlovian conditioning procedures result in dramatic individual differences in the topography of learnt behaviors in rats: When the temporary insertion of a lever into an operant chamber is paired with food pellets, some rats (known as sign-trackers) predominantly interact with the lever, while others (known as goal-trackers) predominantly approach the food well. Two experiments examined the sensitivity of these two behaviors to changing reinforcement contingencies in groups of male and female rats exhibiting the different phenotypes (i.e., sign-trackers and goal-trackers). In both phenotypes, behavior oriented to the food well was more sensitive to contingency changes (e.g., a reversal in which of two levers was reinforced) than was lever-oriented behavior. That is, the nature of the two behaviors differed independently of the rats in which they were manifest. These results indicate that the behavioral phenotypes reflect the parallel operation of a stimulus–stimulus associative process that gives rise to food-well activity and a stimulus–response process that gives rise to lever-oriented activity, rather than the operation of a single process (e.g., stimulus–stimulus) that generates both behaviors.

Pavlovian conditioning is perhaps the most well-known psychological phenomenon, and its theoretical importance was evident from an early point. Reflecting on his extensive research, Pavlov (1928) stated that “experiments with conditioned reflexes have provided associative psychology, i.e., such psychology as believes the association to be the foundation of psychic activity, with a firm basis” (p. 171). The use of his paradigm is widespread, particularly across the fields of behavioral and cognitive neuroscience (for a recent review, see Murphy & Honey, 2016) and behavioral genetics (e.g., Duvarci, Nader, & LeDoux, 2008; Lonsdorf et al., 2009; Schafe et al., 2000; see also, Amstadter, Nugent, & Koenen, 2009). In the field of behavioral neuroscience, one of its principal uses has been in providing a test bed for formal theories of associative learning, which assume that organisms form associations between the representations of conditioned and unconditioned stimuli (e.g., Mackintosh, 1975; Pearce, 1994; Pearce & Hall, 1980; Rescorla & Wagner, 1972; Wagner, 1981). These stimulus–stimulus (S-S) associations have been contrasted with the formation of stimulus–response (S-R) associations between the processes activated by the stimulus and the motor program for generating a response (Hull, 1943; Spence, 1936, 1937). The idea that two (associative) systems might underpin conditioned behavior has clear counterparts in cognitive neuroscience (e.g., Daw, Gershman, Seymour, Dayan, & Dolan, 2011; Dayan & Berridge, 2014).

Like Pavlov, the formal theories of associative learning identified above appeal to the idea that the memory or representation of one stimulus can come to excite (or to inhibit) the representation of another stimulus through an excitatory (or inhibitory) association formed between them. Unlike Pavlov, however, such theories have eschewed consideration of individual differences in Pavlovian conditioning, apart from insofar as they represent one source of variance in behavioral measures of learning (see Matzel et al., 2003). Briefly, Pavlov proposed that the individual differences in the ‘temperament’ of his experimental animals (dogs) during discrimination learning were reflected in differences in their capacities for excitatory and inhibitory learning; and argued that these differences might provide a useful model for various human pathological conditions (Pavlov, 1928, pp. 373–378). Leaving aside the details of Pavlov’s analysis, the view that there are marked individual differences in simple conditioning has been amply confirmed in more recent experiments with rodents, where the differences are perhaps more striking and well characterized. These differences too have potential translational significance (see Flagel, Akil, & Robinson, 2009; Lovic, Saunders, Yager, & Robinson, 2011).

The critical behavioral observations come from simple autoshaping procedures. For example, hungry rats might be placed in operant chambers where they receive brief presentations of a lever that are paired with the delivery of a reinforcer into a food well (e.g., Patitucci, Nelson, Dwyer, & Honey, 2016). This procedure produces marked individual differences in behavior: some rats predominantly interact with the lever while others approach the food well during the lever presentations. Interacting with the lever—the signal for the impending delivery of the reinforcer—is called sign-tracking (e.g., Hearst & Jenkins, 1974), while approaching the food well is called goal-tracking (e.g., Boakes, 1977). These different phenotypes vary continuously across a given cohort of rodents (see Fitzpatrick et al., 2013): with some rats consistently engaging in either lever- or food-well-oriented behavior across training sessions and others showing patterns of behavior between these extremes. The basis for these different phenotypes is the central issue that is addressed here.

One analysis of individual differences in sign- and goal-tracking behavior can be derived from the assumption that the types of associative structures described above (i.e., S-S or S-R) might be differently represented across individuals (see Patitucci et al., 2016; see also, Lesaint, Sigaud, Flagel, Robinson, & Khamassi, 2014). The general idea that acquired behaviors might be the product of different systems with distinct characteristics has a clear precedent in the context of studies of instrumental conditioning (e.g., Dickinson & Balleine, 2002), and there are two sources of evidence that are consistent with it from studies of phenotypic differences in Pavlovian conditioned responding. First, food-well activity in rats classified as goal-trackers (GTs) declines more rapidly during an extinction procedure than does lever pressing in rats classified as sign-trackers (STs; see Ahrens, Singer, Fitzpatrick, Morrow, & Robinson, 2016). These observations suggest that food-well activity reflected the current status of the relationship between the lever and the reinforcer (i.e., an S-S association), whereas lever-oriented behavior was based on a S-R habit that was more resistant to changes in contingencies. Second, the bias toward approaching the food well relative to lever pressing is positively correlated with the palatability of the reinforcer (Patitucci et al., 2016), and sating rodents with the reinforcer affects conditioned food-well activity but not lever-oriented activity (Morrison, Bamkole, & Nicola, 2015; Patitucci et al., 2016).

The results described in the preceding paragraph are consistent with the general idea that there are two learning systems (S-S and S-R) that operate differently across rats; but there are at least two forms that this analysis could take. For example, while food-well behavior might be the dominant response generated by the S-S system and lever-oriented behavior the dominant response generated by the S-R system, both systems might have the capacity to generate both responses (see Lesaint et al., 2014). If a single system governed all behavior in a given rat then both food-well and lever-press responses should exhibit the characteristic property of that system: When governed by an S-S system, activity directed toward both the lever and the food well will change rapidly in the face of a change in contingencies; whereas when governed by an S-R system both will change relatively slowly. In principle, the accuracy of this prediction could have been assessed by Ahrens et al. (2016); but, they only presented activity directed toward the food-well for GTs and toward the lever for STs. It is not, therefore, possible to assess whether the two forms of response were affected differently in rats classified as GTs or STs. The single-system analysis just outlined is, however, challenged by the following observation: A given rodent can be classified as a GT (or ST) with respect to their behavior on a lever that predicts one reinforcer (e.g., food pellets), but not classified in the same way on another lever that predicts a different reinforcer (e.g., sucrose; Patitucci et al., 2016, Experiment 1). If a single-system (S-S or S-R) governed behavior in a given animal then the patterns of behavior should be consistent across different manipulanda (i.e., the left and right levers).

A simple alternative to the analysis described in the previous paragraph assumes that behaviors directed toward the food well and lever are generated by independent systems (S-S and S-R, respectively) that operate in parallel. This analysis predicts that a given form of response will exhibit the same characteristics independently of whether the animal in which it is observed is classified as a ST or a GT; with food-well activity being derived from the operation of a S-S system and lever-oriented behavior being derived from a S-R system that operate to different degrees in all rodents. The dominant response might be toward the food well in one rodent and lever in another, but in both rats food-well activity should more rapidly track changes in reinforcement contingencies than should lever-oriented activity. As already noted, this prediction was not assessed by Ahrens et al. (2016); but Patitucci et al. (2016, Experiment 2) reported that satiation had a marked effect on food-well activity when the effect of this manipulation was considered across rats that had been classified as GTs or STs. This observation is consistent with the idea that activity directed toward the food-well and lever have the same properties irrespective of whether they were exhibited in STs or GTs.^1^

To summarize, to the best of our knowledge nobody has directly investigated the following simple question: Does a given type of behavior (e.g., lever oriented) have the same or different characteristics when assessed in STs and GTs? Here, we addressed this question in two experiments. In both experiments, rats received training procedures that should allow the two phenotypes to develop (cf. Patitucci et al., 2016), and then the contingencies were changed (e.g., the reinforced lever became nonreinforced and vice versa; cf. Ahrens et al., 2016). The changes in behaviors directed toward the lever and food well were then assessed as a function of whether the rodents had been classified as STs or GTs at the end of the first stage of training. Evidence favoring the claim that the S-S system generates food-well activity and the S-R system generates lever-oriented behavior would take the form of a compelling dissociation: More rapid changes in food-well activity than in lever-oriented behavior at the quite different levels of performance anticipated in rats classified as predominantly goal-tracking or sign-tracking (cf. Lesaint et al., 2014).

## Experiment 1

Two levers (L_1_ and L_2_) were inserted into the experimental chambers for 10s on separate trials. Presentations of L_1_ (e.g., the left lever) were immediately followed by the delivery of a single food pellet and presentations of L_2_ (e.g., the right lever) were not (see Table 1). Interactions with the levers and the food-well were automatically recorded, and once a stable level of the two responses was established, the rats were classified as STs and GTs on the basis of whether their behavior was predominantly oriented toward the lever or food well, respectively. The contingencies between the levers and their outcomes were then reversed: L_1_ was followed by no food, and L_2_ was paired with food pellets. The principal issue was the impact of the changed contingencies on the behavior of Groups ST and GT. If the behavior of Group ST is based on a S-R system whereas in Group GT it is based on a S-S system, then both lever presses and food-well entries should change less rapidly in Group ST than in Group GT when the contingencies are reversed. However, if behavior directed toward the lever is based on a S-R system and behavior directed toward the food well is based on a S-S system, then lever-oriented behavior should be less sensitive to the change in contingencies than should food-well behavior in both Groups ST or GT. We also conducted complementary analyses in which the two types of response (lever presses and food-well entries) were treated in a continuous fashion, which provided an additional assessment of the individual consistency in the two behaviors across (1) the final two blocks of training and (2) the final block of training and the first reversal block.[Table-anchor tbl1]

### Method

#### Subjects

Sixteen female Sprague–Dawley rats were used (supplied by Charles River, U.K.). They had been subjects in a behavioral task involving drinking different concentrations of sucrose, but were naïve with respect to the apparatus and procedures used in Experiment 1.^2^ The rats were housed in groups ranging from two to four in standard cages and maintained on 12-hr/12-hr light/dark cycle (lights on at 7 a.m.). Their mean ad libitum weight before the start of the experiment was 321g (range = 280–366 g) and they were maintained at between 85 and 95% of these weights by giving them restricted access to food at the end of each day. The rats had continuous access to water when they were in their cages. The research was conducted in accordance with Home Office regulations (Animals [Scientific Procedures] Act 1986, 1990).

#### Apparatus

Eight identical conditioning boxes measuring 30 × 24 × 21 cm (height × width × depth; Med Associates, Georgia, VT) were used. Each box was placed in a sound-attenuating shell that incorporated a ventilation fan, which maintained the background noise at 68 dB(A). The boxes had aluminum side walls and clear acrylic front, back and top. The floor was constructed from 19 steel rods (4.8 mm diameter, 16 mm apart) and was situated above a stainless steel tray. Food pellets (45 mg, supplied by MLab, Richmond, IN) were delivered to a floor-level recessed food well (aperture: 5.3 × 5.3 cm) in the center of the left wall. The food well was equipped with infrared detectors that allowed the presence of the rat in the well to be automatically recorded. A single response was registered when the detector was interrupted (e.g., when a rat’s snout entered the food well). Two retractable levers (4.5 × 1.8 × 0.2 cm) were located 3 cm to the left and right of the food well and at a height of 4.6 cm and 1.5 cm from the edge of the wall. A lever press was recorded each occasion that the lever was depressed by 4 mm from its usual horizontal resting position. MED-PC software was used to insert levers, deliver food pellets, and to record food well entries and lever presses.

#### Procedure

The rats had two 24-min pretraining sessions when food pellets were delivered on a variable-time 60-s schedule (range = 40–80 s). Rats then received a single session of training on each of the next 12 days of training, which occurred at the same time of day for a given rat. These sessions consisted of 20 trials on which the left lever was inserted for 10 s and then retracted and 20 trials on which the right lever was inserted for 10 s and was then withdrawn. For half of the rats, the retraction of the left lever was immediately followed by the delivery of one food pellet and the right lever was not reinforced; and for the other half the right lever was reinforced and the left lever was not. The order in which the left and right levers were presented was random with the constraint that there could not be more than three same type trials in succession. The trials were delivered on a variable-time 60-s schedule (range = 40–80 s). All rats then received reversal training for 12 days in which L_1_ (e.g., left lever) was not followed by food and L_2_ (e.g., right lever) was reinforced. The fact that the identities of the levers that served as L_1_ and L_2_ during training was counterbalanced means that the identities of the levers that served as L_1_ and L_2_ during reversal was also counterbalanced. The procedure used for the reversal stage was in other respects identical to the training stage.

#### Data analysis

Successive sessions during the training and reversal stages were combined into twelve 2-day blocks (six training: T1–T6; and six reversal: R1–R6). At the end of the training phase, the rats were split into two groups (*n* = 8 in both groups), STs and GTs, based on their tendency to engage with the lever and the food well. A bias score was calculated using the number of lever presses and food-well entries for the reinforced lever, L_1_: (goal-tracking − sign-tracking)/(goal-tracking + sign-tracking). A median split was used to divide rats into those with higher scores (Group GT for GTs) and those with lower scores (Group ST for STs). Subsequent analyses were conducted separately for lever presses and food-well entries, with the main focus being on the transition between the final block of training (T6) and the first block of reversal (R1). These analyses used SPSS Statistics (Version 23) and RStudio (R Development Core Team, 2008) with Greenhouse–Geisser correction when necessary. As already mentioned, we also conducted complementary analyses in which the number of lever presses and food-well entries were treated in a continuous fashion.

Standard hypothesis testing does not directly assess whether the absence of a significant effect is sufficient evidence to conclude that there is no effect. In contrast, Bayesian statistics provides a ratio of the probability for the observed data under different models, such as a model based on the null hypothesis relative to a model based on some specified alternative model. The resulting Bayes factors can then be interpreted according to the convention suggested by Jeffreys (1961; see also, Rouder, Speckman, Sun, Morey, & Iverson, 2009) where a Bayes factor between 1 and 3 provides anecdotal support, a factor between 3 and 10 suggests some supporting evidence, while a factor beyond 10 indicates strong evidence. If lever-pressing or food-well activities are not differently affected by the reversal then our inferences are based on classical tests not being significant, without being able to draw any conclusions about the null hypothesis. We have, therefore, supplemented standard null-hypothesis statistical testing with the presentation of equivalent Bayes factors, when null results are of theoretical significance. Bayesian analysis was conducted using JASP software (Version 0.8.1.2) with Bayes factors for main effects and interactions for factorial analysis of variance ANOVA as described by Rouder, Morey, Speckman, and Province (2012) and Rouder, Morey, Verhagen, Swagman, and Wagenmakers (2017).

### Results

**Figure 1 fig1:**
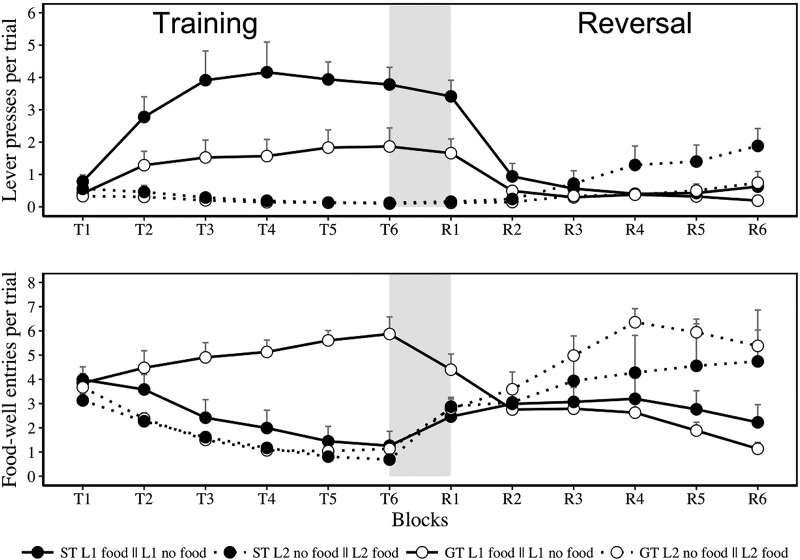
Experiment 1. Mean (+*SEM*) lever presses (upper panel) and food-well entries (lower panel) per (10-s) trial across the two stages: training (T1–T6) and reversal (R1–R6). During training, rats received presentations of one lever paired with food pellets (L_1_-food) and nonreinforced presentations of a second lever (L_2_-no food); rats were classified as sign-trackers (STs) and goal-trackers (GTs) on the basis of their behavior during the final block of training (T6). They then received a reversal: L_1_-no food and L_2_-food. The gray section indicates transition between initial training and the reversal of the contingencies. The means for L_1_ in Group ST, for example, across the training (L_1_-food) and reversal (L_1_-no food) stages, are denoted ST L1 food || L1 no food.

The principal results from Experiment 1 are shown in Figure 1, with lever presses in the upper panel and food-well entries in the lower panel. Our analysis will begin with results from the training stage, before moving to the critical transition between training and reversal (identified by the gray section), and finally the reversal stage as a whole.[Fig-anchor fig1]

#### Training

Inspection of the results from the first stage of training (left-hand side of the upper and lower panels) suggests that as training progressed rats in both groups (ST and GT) showed more lever presses and food-well entries during the reinforced L_1_ than the nonreinforced L_2_. The fact that during the initial training sessions there was a higher level of food-well entries than lever presses probably reflects the impact of the pretraining sessions in which food pellets were delivered into the food well. In any event, the discrimination involving lever presses was more evident in Group ST than Group GT, while the discrimination involving food-well entries was more evident in Group GT than Group ST, with these between-groups differences being most apparent on reinforced L_1_ trials. The description of the training results is supported by separate analyses of lever presses and food-well entries. ANOVA conducted on lever presses confirmed that there were main effects of group (ST vs. GT), *F*(1, 14) = 6.35, *p* = .024, η_p_^2^ = .31, lever (L_1_ vs. L_2_), *F*(1, 14) = 40.05, *p* < .001, η_p_^2^ = .74, and block (1–6), *F*(2.96, 41.41) = 9.74, *p* = .001, η_p_^2^ = .41. There was no interaction between group and block, *F*(2.96, 41.41) = 2.15, *p* = .109, η_p_^2^ = .13, but there was an interaction between lever and block, *F*(2.60, 36.35) = 18.74, *p* = .001, η_p_^2^ = .57, between group and lever, *F*(1, 14) = 6.88, *p* = .020, η_p_^2^ = .33, and a three-way interaction between group, lever and block, *F*(2.60, 36.35) = 3.41, *p* = .033, η_p_^2^ = .19. A parallel analysis of food-well entries revealed a main effect of group, *F*(1, 14) = 12.19, *p* = .004, η_p_^2^ = .46, lever, *F*(1, 14) = 37.81, *p* < .001, η_p_^2^ = .73, and block, *F*(2.68, 37.49) = 11.98, *p* < .001, η_p_^2^ = .46. There were also interactions between block and lever, *F*(2.83, 39.69) = 7.40, *p* < .001, η_p_^2^ = .34, group and block, *F*(2.68, 37.49) = 7.29, *p* < .001, η_p_^2^ = .34, and between group and lever, *F*(1, 14) = 12.89, *p* = .003, η_p_^2^ = .48. The three-way interaction was also significant, *F*(5, 70) = 10.33, *p* < .001, η_p_^2^ = .42.

#### Transition between training and reversal stages

Of central interest are the results from the transition between the final block of training and first block of reversal (identified in the gray section of Figure 1). Inspection of this transition highlights the fact that lever presses remained stable in spite of the reversed reinforcement contingencies (upper panel), whereas food-well entries changed rapidly (lower panel). Moreover, these differences between the effects of the reversal on lever presses and food-well entries were evident in both Groups ST and GT: The levels of lever pressing remained largely unchanged in both groups; and while there was a marked decrease in food-well entries to the previously reinforced lever in Group GT there were marked increases in food-well entries to the previously nonreinforced lever in both Group GT and ST. Also, in Group ST there was a more marked increase in responding between the T6 and R1 for L_2_ than L_1_.

Separate analyses conducted on lever presses and food-well entries for the final block of training (i.e., T6) with the first block of reversal training (i.e., R1) confirmed the description presented in the immediately preceding paragraph. ANOVA conducted on lever presses during T6 and R1 revealed that there was an effect of group (ST or GT), *F*(1, 14) = 7.71, *p* = .015, η_p_^2^ = .35, lever (L_1_ or L_2_), *F*(1, 14) = 55.27, *p* < .001, η_p_^2^ = .79, and no effect of block, *F*(1, 14) = 1.67, *p* = .216, η_p_^2^ = .1. There was an interaction between group and lever, *F*(1, 14) = 6.90, *p* = .02, η_p_^2^ = .33, but critically there was no interaction between block and lever, group and block, and no three-way interaction (both *F*s <1). The Bayes factor for the best model without the interaction between block and lever relative to the model with the interaction was 9.52, which indicates evidence against the presence of the interaction. That is, the difference between L_1_ and L_2_ remained unchanged despite the reversal of reinforcement contingency. The Bayes factor for the best model without the interaction between block, lever and group relative to the model with the interaction was 62.50, which represents strong evidence against the presence of the three-way interaction. That is, Groups ST and GT did not differ in terms of the (absence of) a Lever × Block interaction.

In marked contrast, a parallel analysis of food-well entries during T6 and R1 revealed that the reversal had an immediate effect. There was a main effect of group, *F*(1, 14) = 18.90, *p* = .001, η_p_^2^ = .57, lever, *F*(1, 14) = 11.88, *p* = .004, η_p_^2^ = .45, and block, *F*(1, 14) = 12.03, *p* = .004, η_p_^2^ = .46. More importantly, there was an interaction between lever and block, *F*(1, 14) = 43.67, *p* < .001, η_p_^2^ = .75, as well as between group and block, *F*(1, 14) = 9.68, *p* = .008, η_p_^2^ = .4, group and lever, *F*(1, 14) = 11.88, *p* = .005, η_p_^2^ = .43, and a three-way interaction between group, block and lever, *F*(1, 14) = 11.86, *p* = .004, η_p_^2^ = .45. Separate analysis of the scores from Group GT revealed a main effect of lever, *F*(1, 7) = 18.48, *p* = .004, η_p_^2^ = .72, but no effect of block (*F* < 1), and an interaction between block and lever, *F*(1, 7) = 35.37, *p* = .001, η_p_^2^ = .835. An equivalent analysis of Group ST revealed a main effect of block, *F*(1, 7) = 46.83, *p* < .001, η_p_^2^ = .87, but not of lever, *F*(1, 7) = .018, *p* = .896, η_p_^2^ = .003, and an interaction between block and lever, *F*(1, 7) = 8.70, *p* = .021, η_p_^2^ = .556. Food-well entries were sensitive to the reversal of the reinforcement contingencies in both the ST and GT groups.

The analysis just presented involved dividing rats into two groups (ST and GT) using their biases during the final block of training. However, the same conclusions are supported by an analysis in which their lever presses and food-well entries are treated as a continuum.

**Figure 2 fig2:**
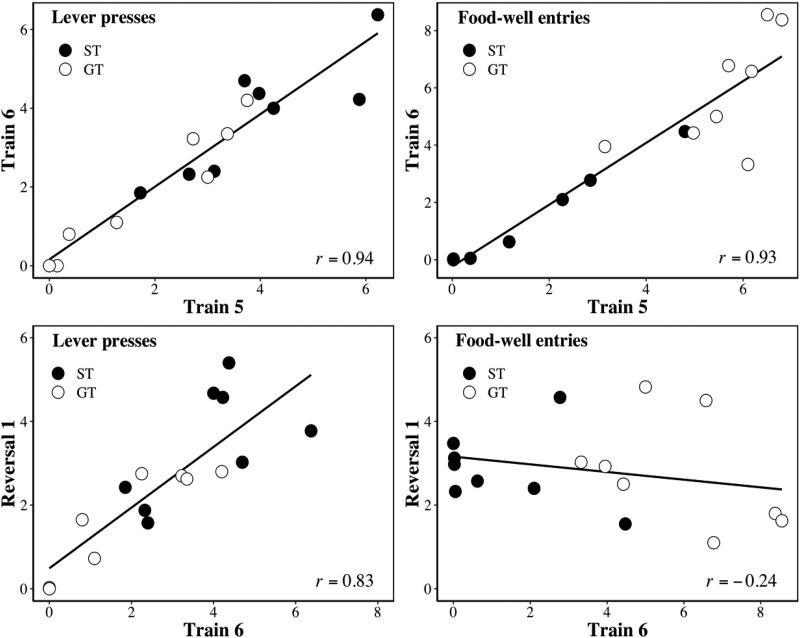
Experiment 1. The upper panels show the relationship between the mean number of responses per (10-s) trial between blocks T5 and T6 for lever presses (left-hand panel) and for food-well entries (right-hand panel) on reinforced L_1_ trials. The lower panels show the relationship T6 and R1 for lever presses (left) and food-well entries (right) on L_1_ trials. The closed symbols correspond to rats classified as sign-trackers (i.e., Group ST) and the open symbols to those classified as goal-trackers (i.e., Group GT).

The upper panels of Figure 2 depict the relationship between lever presses on reinforced L_1_ trials for the final blocks of training (i.e., T5 and T6; left-hand panel), and between food-well entries on L_1_ trials for the same blocks (right-hand panel). The lower panels depict the relationships between lever presses on the final block of training and the first block of reversal (i.e., T6 and R1 left-hand panel) on L_1_ trials, and between food-well entries for the same two blocks (right-hand panel) on L_1_ trials. The group membership of each rat is identified. For both types of response, there was a significant correlation between T5 and T6 for lever presses, *r*(14) = .94, *p* < .001 (lever presses) and *r*(14) = .93, *p* < .001 (food-well entries). However, while there was a correlation between T6 and R1 for lever presses, *r*(14) = .83, *p* < .001, there was not for food-well entries, *r*(14) = −.24, *p* = .355. Food-well entries changed between T6 and R1, but lever presses did not.[Fig-anchor fig2]

#### Reversal

Across the blocks of reversal training, the numbers of lever presses increased during L_2_ and decreased during L_1_ in Group ST, and this change was less evident in Group GT. In contrast, the number of food-well entries increased during L_2_ and decreased in L_1_ in Group GT, and this change was less apparent in Group ST. ANOVA performed on lever presses during the reversal confirmed that there was a main effect of block (1–6), *F*(2.77, 38.84) = 17.62, *p* < .001, η_p_^2^ = .55, and no effect of group (ST vs. GT), *F*(1, 14) = 3.79, *p* = .072, η_p_^2^ = .21, or lever (L_2_ vs. L_1_), *F*(1, 14) = 1.18, *p* = .295, η_p_^2^ = .07. There was an interaction between lever and block, *F*(5, 70) = 21.67, *p* < .001, η_p_^2^ = .6, no interaction between group and lever, *F* < 1, and a three-way interaction between group, lever and block, *F*(1.68, 23.51) = 3.73, *p* = .046, η_p_^2^ = .21. There was no interaction between group and block, *F*(2.77, 38.84) = 2.53, *p* = .076, η_p_^2^ = .15. A parallel analysis of food-well entries revealed a main effect of lever, *F*(1, 14) = 23.05, *p* < .001, η_p_^2^ = .62, and no effect of group, *F*(1, 14) = 1.67, *p* = .693, η_p_^2^ = .01, or block, *F*(1.70, 23.77) = 1.58, *p* = .176, η_p_^2^ = .1. There was also an interaction between block and lever, *F*(1.87, 26.12) = 13.47, *p* < .001, η_p_^2^ = .49, but no interaction between either group and block or group and lever (largest *F*(1, 14) = 2.56, *p* = .132, η_p_^2^ = .15). The three-way interaction was again significant, *F*(1.87, 26.12) = 4.05, *p* = .003, η_p_^2^ = .22.

### Discussion

Discrimination training where the presentation of one lever (L_1_) was paired with food pellets and another (L_2_) was not, resulted in marked individual differences in conditioned responding; with some rats interacting with L_1_ (but not L_2_) and others approaching the site of food delivery during L_1_ (but not L_2_; see also Patitucci et al., 2016). When the contingencies were reversed, with L_1_ now nonreinforced and L_2_ reinforced, the different levels of lever pressing to L_1_ (and L_2_) in Groups ST and GT remained remarkably stable during the first block of reversal. In contrast, the levels of food-well entries changed more rapidly in both Groups ST and GT (see Figure 1). This differential sensitivity of the two response forms to changing contingencies was also evident when they were considered as continuous variables (see Figure 2). These results demonstrate that the dissociation between lever presses (in rats designated as STs) and food-well entries (in rats designated as GTs) does not reflect a difference in the sensitivity of the two groups to changed reinforcement contingencies per se (cf. Ahrens et al., 2016). Instead, these results show that the lever press and food-well entry responses are differently sensitive to such changes irrespective of the phenotype of the rat. These observations suggest that the distinct behaviors reflect the parallel operation of S-S and S-R systems within an individual, rather than the operation of a single system (either S-S or S-R) that gives rise to both behaviors (cf. Lesaint et al., 2014). Experiment 2 attempted to extend these observations by examining whether changes in the nature of the reinforcer (between alternatives that produce different levels of responding) produce more rapid changes in food-well activity than in lever pressing in STs and GTs.

## Experiment 2

Rats received separate presentations of two levers (L_1_ and L_2_) that were both paired with the same reinforcer during training (either food pellets or sucrose). Pilot research had established that food pellets maintain higher levels of both lever pressing and food-well entries than does sucrose (see also, Patitucci et al., 2016); which should be evident in the first stage of training in Experiment 2. The rats that were reinforced with pellets and sucrose were further divided into two groups (Group ST ad GT) on the basis of their biases at the end of training. During the second stage, the reinforcers associated with the two levers were switched: the rats given pellets during training received sucrose during the switch and those given sucrose during training received food pellets during the switch (see Table 2). The issue of central interest was the extent to which the two target behaviors that had developed during L_1_ and L_2_ (lever presses and food-well entries) would change to reflect the fact that the levers were now paired with reinforcers that maintained different levels of performance (i.e., food pellets and sucrose). If the behavior of rats in Group ST is generated by a S-R system, then both lever presses or food-well entries should be less sensitive to the change in reinforcer type than those in Group GT, whose behavior is generated by a S-S system. However, if lever pressing is based on a S-R system whereas food-well entries reflect a S-S system, then lever pressing should be less sensitive to the change in contingencies between the training and switch stages than should food-well entries, irrespective of whether those behaviors are expressed in Group ST or Group GT.^3^

### Method

#### Subjects and apparatus

Thirty-two naïve male (outbred) Lister Hooded rats (supplied by Envigo, Bicester, U.K.) were housed in groups of four in standard cages and maintained on 12-hr/12-hr light/dark cycle (lights on at 7 a.m.). Their mean ad libitum weight was 295 g (range = 284–320 g). Rats had free access to water and they were maintained between 85 and 95% of their ad lib weights by giving them restricted access to food at the end of each day. The experimental chambers were those used in Experiment 1, but in Experiment 2 the sucrose dipper delivered 0.05 ml of sucrose solution (8% weight/weight with water). When sucrose was scheduled to be delivered, the dipper was immersed in the sucrose and then raised back into the reward well.

#### Procedure

The rats had two 24-min pretraining sessions before the training and switch stages. During these pairs of sessions, the rats received the reinforcer (food pellets or sucrose) that was to be delivered in the immediately succeeding stage. The reinforcers were delivered on a variable-time 60-s schedule (range = 40–80 s). Rats received 12 days of training that were arranged in the same way as Experiment 1 with the exception that the presentation of both levers (L_1_ and L_2_) were followed by a reinforcer (food pellets for half of the rats and sucrose for the remainder). The switch stage also consisted of 12 days. This stage was identical to the training stage with the exception that the rats that had received food pellets during the training stage received sucrose during the switch stage, and those that had received sucrose during training received food pellets during the switch.

#### Data analysis

There were strong positive correlations between lever press behavior during the presentations of L_1_ and L_2_ that were both paired with the same outcome (either food pellets or sucrose) and between food well behavior on the two levers. These observations have some theoretical significance when contrasted with the results of Patitucci et al. (2016), who reported no correlation between the sign- and goal-tracking biases on two levers that signaled different outcomes (i.e., L_1_-food pellets and L_2_-sucrose). We shall consider the implications of this evidence in the General Discussion. However, to simplify the results section, the principal analysis of the results of Experiment 2 will be conducted with the frequency of responses combined across the left and right levers. As in Experiment 1, the training and switch sessions were combined into 2-day blocks for the purpose of analysis. The rats were split into two groups, STs and GTs, using the bias score described in Experiment 1. The split was conducted separately for the subgroups of rats that received food pellets and sucrose during the training stage. This resulted in four groups (*n* = 8 for each group): ST Pellets || Sucrose (STs; pellets during training, sucrose during the switch stage), GT Pellets || Sucrose (GTs; pellets during training, sucrose during the switch), ST Sucrose || Pellets (STs; sucrose during training, pellets during the switch) and GT Sucrose || Pellets (GTs; sucrose during training, pellets during the switch).

### Results

**Figure 3 fig3:**
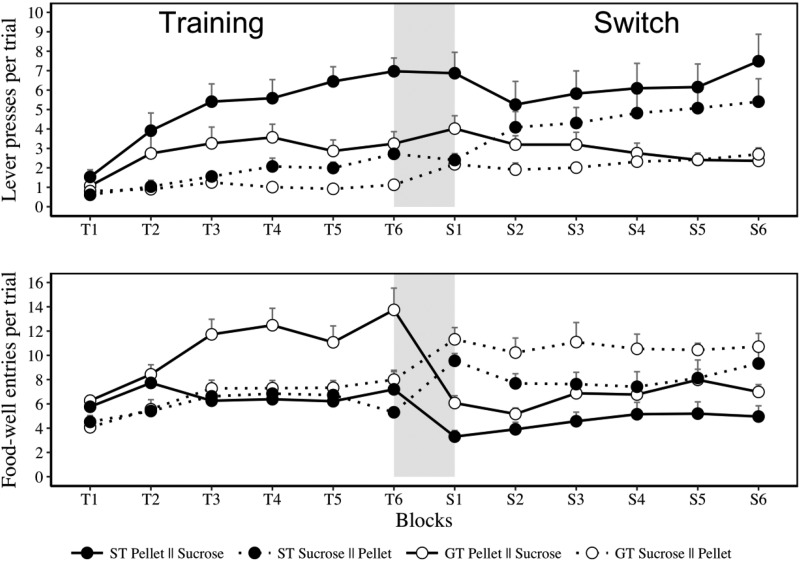
Experiment 2. Mean (+*SEM*) lever presses (upper panel) and food-well entries (lower panel) per (10-s) trial across the two stages: training (T1–T6) and switch (S1–S6). During training, rats received presentations of two levers (L_1_ and L_2_) paired with either pellets or sucrose. Rats were classified as sign-trackers (ST) and goal-trackers (GT) on the basis of their behavior during the final block of training (T6). The reinforcers that followed the levers were transposed during the second switch stage. The gray section indicates transition between initial training and the swap from sucrose to food pellet rewards (or from pellets to sucrose). For example, the means for the ST group that received food pellets during training and sucrose during the switch are given by the label ST Pellet || Sucrose.

The main results from Experiment 2 are shown in Figure 3, with lever presses in the upper panels and food-well entries in the lower panels. As in Experiment 1, our analysis of the results of Experiment 2 will begin with results from the training stage, before moving to a comparison of the final block of training with the first block of reversal (identified by the gray section), and finally the switch stage as a whole.[Fig-anchor fig3]

#### Training

Inspection of the left-hand side of the upper and lower panels in Figure 3 suggests that the ST groups are more likely to engage in lever pressing than are the GT groups, and that the GT groups are more likely to enter the food well than the ST groups. These group differences, especially in the case of food-well activity, were most marked when food pellets were the reinforcer. This description of the training results presented in Figure 3 is supported by separate analyses of lever presses and food-well entries. ANOVA conducted on lever presses, pooled across the two levers, confirmed that there were main effects of group (ST or GT), *F*(1, 28) = 9.65, *p* = .004, η_p_^2^ = .25, reinforcer (food pellets or sucrose), *F*(1, 28) = 31.01, *p* < .001, η_p_^2^ = .52, and block (1–6), *F*(3.15, 88.33) = 24.96, *p* < .001, η_p_^2^ = .47. There were interactions between group and block, *F*(3.15, 88.33) = 7.11, *p* < .001, η_p_^2^ = .2, and reinforcer and block, *F*(3.15, 88.33) = 7.76, *p* < .001, η_p_^2^ = .21, but no group and reinforcer interaction, *F*(1, 28) = 2.75, *p* = .108, η_p_^2^ = .09. The three-way interaction, *F*(3.15, 88.33) = 1.05, *p* = .373, η_p_^2^ = .03, was not significant. A parallel analysis of food-well entries revealed that there were main effects of group (ST or GT), *F*(1, 28) = 9.91, *p* = .004, η_p_^2^ = .26, reinforcer (food pellets or sucrose), *F*(1, 28) = 9.94, *p* = .004, η_p_^2^ = .26, and block (1–6), *F*(2.87, 80.34) = 19.10, *p* < .001, η_p_^2^ = .41. There were interactions between group and reinforcer, *F*(1, 28) = 5.00, *p* = .033, η_p_^2^ = .15, group and block, *F*(5, 140) = 9.38, *p* < .001, η_p_^2^ = .25, and no interaction between reinforcer and block, *F*(3.15, 88.33) = 1.93, *p* = .133, η_p_^2^ = .06. There was also a three-way interaction, *F*(2.87, 80.34) = 3.28, *p* = .027, η_p_^2^ = .11.

#### Transition between training and reversal stages

Inspection of the gray panel in Figure 3 shows that there were rapid changes in food-well entries (lower panel) but not in lever pressing (upper panel). To be more specific: The high level of food-well activity—previously maintained by pellets—declined, and the low level of food-well activity—previously maintained by sucrose—increased. In contrast, lever pressing was largely unchanged across T6 and S1. This description was supported by separate analysis of lever presses and food well entries. ANOVA conducted on lever presses revealed an effect of group, *F*(1, 28) = 14.71, *p* = .001, η_p_^2^ = .34, an effect of reinforcer, *F*(1, 28) = 33.57, *p* < .001, η_p_^2^ = .54, and no effect of block, *F*(1, 28) = 1.86, *p* = .183, η_p_^2^ = .06. There was an interaction between group and block, *F*(1, 28) = 4.69, *p* = .039, η_p_^2^ = .144. Critically, there was no interaction between block and reinforcer and no three-way interaction (*F*s < 1). The Bayes factor for the best model without the block by lever interaction relative to the best model with the interaction is 5.88, indicating evidence against the presence of the interaction. The Bayes factor for the best model without the Block × Lever × Group interaction relative to the model with the interaction is 100, indicating strong evidence against the presence of the interaction. The switch in reinforcer had little impact on lever press behavior in either the ST or GT groups.

In contrast, a parallel analysis of food-well entries revealed that the switch had an immediate effect in Groups GT and ST. This analysis confirmed that there was a main effect of group, *F*(1, 28) = 15.59, *p* < .001, η_p_^2^ = .35, and block, *F*(1, 28) = 4.18, *p* = .05, η_p_^2^ = .13, but no effect of reinforcer, *F*(1, 28) = 1.22, *p* = .278, η_p_^2^ = .04. Critically, there was an interaction between reinforcer and block, *F*(1, 28) = 95.42, *p* < .001, η_p_^2^ = .77, as well as between group and block, *F*(1, 28) = 5.75, *p* = .023, η_p_^2^ = .17, but no interaction between group and reinforcer, *F*(1, 28) = 1.94, *p* = .174, η_p_^2^ = .06, and no three-way interaction, *F*(1, 28) = 2.13, *p* = .155, η_p_^2^ = .07. The Bayes factor for the best model without the interaction between block, lever and group relative to the model with the interaction is 25, indicating strong evidence against the interaction. The switch in reinforcers had an immediate impact on behavior directed to the food-well, and this was equivalent in both the ST and GT groups. The same conclusions are supported by an analysis in which lever presses and food-well entries were treated as a continuum.

**Figure 4 fig4:**
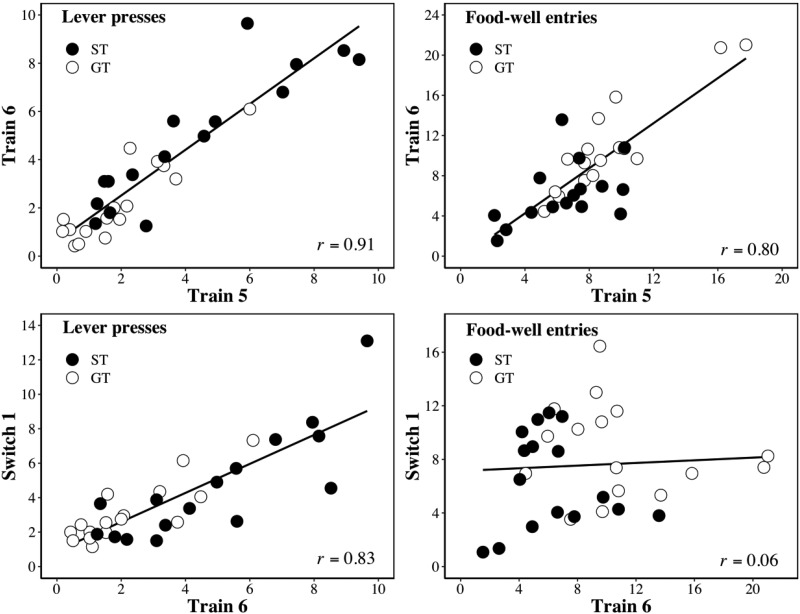
Experiment 2. The upper panels show the relationship between the mean number of responses per (10-s) between blocks T5 and T6 for lever presses (left-hand panel) and food-well entries (right-hand panel) pooled across L_1_ and L_2_ trials. The lower panel shows the relationship T6 and R1 for lever presses (left) and food-well entries (right) pooled across L_1_ and L_2_ trials. The closed symbols correspond to sign-trackers (i.e., Group ST) and the open symbols to goal-trackers (i.e., Group GT).

The upper panels of Figure 4 depict the relationship between lever presses on the final blocks of training (i.e., T5 and T6; left-hand panel) and between food-well entries on the same blocks (right-hand panel) pooled across L_1_ and L_2_ trials. The lower panels depict the relationships between lever presses on the final block of training and the first block of switch (i.e., T6 and S1; left-hand panel), and between food-well entries on the same two blocks (right-hand panel) pooled over L_1_ and L_2_ trials. The group membership of each rat is identified. For both types of response, there was a significant correlation between T5 and T6, *r*(30) = .91, *p* < .001 (lever presses), and *r*(30) = .80, *p* < .001 (food-well entries). Between T6 and S1 there is a significant correlation for lever presses, *r*(30) = .83, *p* < .001, but not for food-well entries, *r*(30) = .06, *p* = .734.[Fig-anchor fig4]

#### Switch

The pattern of results evident on the first block of the switch (i.e., S1) was, for the most part, evident across the later blocks of the switch stage. More specifically, the marked changes in food-well entries were sustained across the switch stage and were accompanied by little change in lever pressing: while the low level of lever pressing increased when sucrose was replaced with food pellets during the switch, the high level of lever pressing was maintained when food pellets were replaced with sucrose. ANOVA conducted on lever presses, pooled across levers, confirmed that there was a main effect of group (ST vs. GT), *F*(1, 28) = 13.69, *p* = .001, η_p_^2^ = .32, no effect of block (1–6), *F*(3.51, 98.17) = 2.19, *p* = .083, η_p_^2^ = .07, and no effect of reinforcer (food pellets or sucrose), *F*(1, 28) = 3.35, *p* = .078, η_p_^2^ = .1. There was an interaction between group and block, *F*(3.51, 98.17) = 4.52, *p* = .003, η_p_^2^ = .14, and reinforcer and block, *F*(3.51, 98.17) = 5.62, *p* < .001, η_p_^2^ = .16, but no interaction between group and reinforcer, and no three-way interaction between group, reinforcer and block (largest *F*(3.51, 98.17) = 1.98, *p* = .112, η_p_^2^ = .07). A parallel analysis of food-well entries revealed a main effect of group, *F*(1, 28) = 6.90, *p* = .014, η_p_^2^ = .19, reinforcer, *F*(1, 28) = 20.46, *p* < .001, η_p_^2^ = .42, and block, *F*(2.87, 80.25) = 3.58, *p* = .019, η_p_^2^ = .11. There was also an interaction between reinforcer and block, *F*(2.87, 80.25) = 5.35, *p* < .005, η_p_^2^ = .16, but no interactions between group and reinforcer or group and block, and no three-way interaction, largest *F*(5, 140) = 1.40, *p* = .225, η_p_^2^ = .04.

### Discussion

The results of Experiment 2 confirm the principal conclusions derived from the results of Experiment 1. First, lever-press behavior was less sensitive to changes in reinforcement contingencies than was food-well behavior. Second, this difference in sensitivity was equally apparent in rats that were classified as STs and GTs. In Experiment 1, these conclusions were supported by the effects of a reversal between the relationships between two levers (L_1_ and L_2_) and the presence and absence of food pellets, whereas in Experiment 2 they were supported by the substitution of reinforcers that maintained more (pellets) or less (sucrose) behavior. The facts that Experiment 2 used male rats while Experiment 1 used female rats (and its results have been replicated in male rats) and the two experiments used different strains (Sprague-Dawley and Lister Hooded, respectively), suggests that the difference in sensitivity of lever and food-well directed behavior to changes in reinforcement contingencies is preserved across rat strains and male/female animals.

## General Discussion

During appetitive Pavlovian conditioning, rodents will reliably display behavior directed both toward the stimulus (sign-tracking) and toward the site of food pellet delivery (goal-tracking). Although individual differences in conditioned responding have typically received scant consideration by theories of associative learning, it is clear that the distribution of these behaviors differs across individuals (e.g., Fitzpatrick et al., 2013). For example, when a lever is temporarily inserted into a conditioning chamber and paired with food pellets some rats develop a consistent tendency to interact with the lever whereas others develop a tendency to approach the food well. These behaviors are differently sensitive to the current value of the reinforcer and indeed its presence. Patitucci et al. (2016) demonstrated that the bias toward engaging in food-well activity rather than lever-press activity was positively correlated with the palatability of the reinforcer; and sating rats on the reinforcer reduced food-well but not lever-oriented activity (see also, Morrison et al., 2015); and Ahrens et al. (2016) showed that lever pressing, in rats that predominantly engaged in sign-tracking, was less sensitive to extinction than food-well activity, in rats that predominantly engaged in goal-tracking. These differences in sensitivity of the two responses are consistent with the involvement of S-R associations in lever-oriented activity and S-S associations in food-well activity (cf. Ahrens et al., 2016; Lesaint et al., 2014).

Our results confirm that lever-press behavior is indeed less sensitive to changes in reinforcement contingencies than is food-well behavior (cf., Ahrens et al., 2016). In Experiment 1, this was evident in the effects of a reversal in the relationships between two levers and the presence and absence of food pellets, whereas in Experiment 2 it was evident in the effects of the substitution of reinforcers that maintained more (pellets) or less (sucrose) behavior. Moreover, in both experiments, these conclusions received additional support from treating lever-press and food-well activity in a continuous way: lever-press activity was correlated between the final block of training (T6) and the first block of the changed contingencies (R1 in Experiment 1 and S1 in Experiment 2), but food-well activity was not.

Here, we contrasted two possible accounts of the behavioral phenotypes. First, that the behavior of a given rodent is governed by the operation of a single system (S-S or S-R), and that the control of both types of behavior simply reflects the nature of the governing system. This account predicts that food-well and lever-oriented behaviors will exhibit different properties in STs and GTs. Second, that behaviors directed toward the food well and lever are generated by independent systems (S-S and S-R, respectively) that operate in parallel. This analysis predicts that a given form of response will exhibit the same characteristics in a STs and GTs. In Experiments 1 and 2, lever-press and food-well behavior in both goal-tracking and sign-tracking rats showed the same pattern of sensitivity to changes in reinforcer contingencies. This pattern of results provides clear support for the second of these accounts: Behaviors directed toward the food well and lever are generated by independent systems (S-S and S-R, respectively) that operate in parallel (cf. Lesaint et al., 2014). Lesaint et al. (2014) also considered the possibility that independent systems contributed to Pavlovian conditioned behavior; arguing that a “model-free” system promotes sign-tracking and a “model-based” system promotes goal tracking. Our data reinforce the general idea that these systems should be thought to operate in parallel. That is, the properties of learnt responses to the lever and food-well are “in the behavior” and not “in the rat,” with individual differences reflecting the fact that S-R and S-S systems are differently weighted across rats rather than there being categorical differences in the learning systems between rats.

While the results that we have presented so far have clear implications regarding the control of behaviors in the two behavioral phenotypes they do not contribute to our understanding the origin of the two phenotypes. Patitucci et al. (2016) argued that food-well activity was more likely to dominate in (goal-tacking) rats that—for whatever reason—valued the reinforcer more. Direct support for this argument, in the form of differences in the palatability of the reinforcer and the effect of reinforcer devaluation, has already been presented in some detail (see also, Morrison et al., 2015; see also, Cleland & Davey, 1982; see also, Davey & Cleland, 1984). But, they also presented some additional evidence that we have only mentioned in passing here. Patitucci et al. (2016, Experiment 1) observed that the classification of a rat as either a GT (or ST) on a lever that was paired with one reinforcer (e.g., food pellets) was unrelated to the classification of the same rat on a second lever that was paired with a different reinforcer (e.g., sucrose). They argued that if a given rat valued one reinforcer (e.g., food pellets) more than the other (e.g., sucrose) then this would result in more goal-tracking on one lever than another. While this analysis is certainly consistent with other features of their results, a more prosaic account can be developed for the lack of correlations between the behaviors directed to two levers: It might have reflected superstitious reinforcement of different behaviors (e.g., lever or food well oriented) that happened to occur during the two levers. However, in Experiment 2, the two levers were both paired with the same reinforcer (food pellets or sucrose), and while this necessarily means that there is no difference in the value of the reinforcer that is paired with the levers, it remains possible that rats will be engaging in different behaviors during the two levers that would be subject to superstitious reinforcement. The results of Experiment 2 provide support for the explanation preferred by Patitucci et al. (2016): When the levers were paired with the same reinforcer there were significant correlations between food-well activity on the left and right levers on Block 6, *r*(30) = .94, *p* < .001, and between lever-press activity on the two levers during the same block, *r*(30) = .74, *p* < .001. The fact that the extent to which phenotypic variation in sign- and goal-tracking behaviors is consistent across levers depends on whether they are paired with the same or different outcomes suggests that outcome value contributes to response selection (cf. Honey, Close, & Lin, 2010).

To summarize, the results presented here and in Patitucci et al. (2016) provide converging support for the view that individual differences in the topography of conditioned behavior reflect the operation of distinct associative processes that differ in their sensitivity to reward value (see also Cleland & Davey, 1982; Davey & Cleland, 1984; Morrison et al., 2015) and changes in contingencies (see also Ahrens et al., 2016). According to this view, a S-S process governs food-well activity, and a S-R process governs lever-press activity. Our results provide the first direct evidence that these systems operate in parallel in a given animal, but are differently weighted between animals.

## Figures and Tables

**Table 1 tbl1:** Design of Experiment 1

Classification	Training	Reversal
ST or GT	L_1_-food and L_2_-no food	L_1_-no food and L_2_-food
*Note*. ST = sign-tracker; GT = goal-tracker. L_1_ and L_2_ refer to two levers (left and right, counterbalanced); food and no food were delivered after the designated lever during training and reversal. Rats were classified as STs or GTs on the basis of their bias toward lever or food-well behavior during the last block of training.		

**Table 2:** Design of Experiment 2

Group	Training	Switch
ST or GT	L_1_-food and L_2_-food	L_1_-sucrose and L_2_-sucrose
ST or GT	L_1_-sucrose and L_2_-sucrose	L_1_-food and L_2_-food
*Note*. ST = sign-tracker; GT = goal-tracker. L_1_ and L_2_ refer to two levers (left and right, counterbalanced). During training, both levers were paired with one reinforcer (food pellets or sucrose), and during the switch, both levers were then paired with the other reinforcer (sucrose or food pellets, respectively). Rats were classified as STs or GTs on the basis of their bias towards lever pressing or entering the food well during the final block of training.		

## Footnotes

1 A supplementary analysis of the food-well activity, where rats were split into groups of sign-trackers (*n* = 8) and goal-trackers (*n* = 8), revealed that there was no interaction between group and satiation, *F*(1,14) = 2.29, *p* = .106, but a Bayesian ANOVA analysis gives a Bayes factor of only 1.64 in favor of the absence of an interaction, which equates to anecdotal/inconclusive evidence with respect to the hypothesis that satiation had an equivalent effect on food-well activity in sign- and goal-tracking rodents.

2 Experiment 1 was replicated as part of a larger study in which the rats were from the same source as Experiment 1, but were male rather than female. The results from this replication matched those of Experiment 1. Experiment 2 also used male rats to confirm the generality of the results from Experiment 1.

3 The relationship between behavior on L_1_ and L_2_, which share a common outcome (in contrast to Patitucci et al., 2016, where L_1_ and L_2_ had different outcomes), is an issue of secondary interest that will be considered in the General Discussion.
